# Is There a Sensitive Period in Human Incest Avoidance?

**DOI:** 10.1177/147470491100900213

**Published:** 2011-04-01

**Authors:** Liqun Luo

**Affiliations:** Research Center for Social Development & Social Policy, Central China Normal University, Wuhan, P. R. China

**Keywords:** incest, early cosocialization, sexual aversion, sensitive period, MPA

## Abstract

Many studies support the proposition that early cosocialization with opposite-sex children has the effect of inhibiting later mutual sexual attraction, but the existence of a period in the life cycle in which individuals are sensitive to the effect of early cosocialization has been a matter of controversy. Drawing on earlier traditional psychological research, and on more recent work guided by parental investment theory, we hypothesized that only for maternal perinatal association (MPA)-absent males a less-than-around-three-years age difference with the sister can predict stronger aversion to sibling incest. The results corroborated the hypothesis. The results can be interpreted as support for the existence of a sensitive period as well as for the potent role of MPA. Cross-cultural comparative studies were called on to further test the hypothesis.

## Introduction

Scholars have increasingly accepted Westermarck's 1891 hypothesis that children who are reared together will later be disinterested in or averse to sex with each other (Westermarck Hypothesis, WH hereafter). However, is there a phase in the life cycle in which individuals are especially sensitive to the effect of early association? In short, is there a sensitive period in human incest avoidance?

Shepher (1971) held that his data about premarital sexual behavior and marriage patterns in Israeli kibbutzim supported the WH. He further argued that early childhood association during the birth to six-years period should be responsible for mutual sexual disinterest or aversion in post-pubescent years. This conclusion about a sensitive period depended, however, on 14 intra-peer group marriages, for eight of which the couples joined the same peer group after the age of six years. Nevertheless, reviewing the data in his Table 3 (p. 297) and following his logic, we can also reach the conclusion that the sensitive period should be ages birth to three years, for 12 out of the 14 couples were in the same peer group only after age three.

According to Wolf (1995, pp. 201-202; 2005), the sensitive period occurs during the first three years of life. His conclusions rely primarily on the fertility of women in minor marriages. The study observed an upward trend in fertility as the age at adoption increased, but noted that fertility changes after adoption later than age three are much smoother. However, Lieberman and Symons (Lieberman, 2007; Lieberman and Symons, 1998) warn that considering Wolf's data requires a “dark lens”, referring to the study's abundance of inferential links between data and conclusions. For instance, the study sought to determine if and how early cosocialization affected later sexual attraction, but data collected about marriages only indirectly measures human psychology.

Fessler and Navarrete (2004) and Lieberman, Tooby and Cosmides (2003a; 2007) directly surveyed living humans to measure variables relevant to incest avoidance. These studies question or deny the existence of a sensitive period. Fessler and Navarrete posit that, if the WH is correct and there is a sensitive period, 1) participants with a smaller age difference between them and their opposite-sex siblings should be exposed to greater cosocialization in a sensitive period and greater cosocialization generally, and thus should exhibit stronger aversion to sibling incest, and 2) participants with older opposite-sex siblings should have a stronger aversion than participants with younger siblings for the same reason. The study generated linear correlations to test the above expectations, but only male participants exhibited a negative relationship between age disparity and sibling incest aversion.

Lieberman, Tooby, and Cosmides (2003b) proposed an evolutionary psychological approach to human kin recognition and human incest avoidance, laying the theoretical foundation for further empirical studies. Lieberman, Tooby, and Cosmides (2007) further suggested a sibling detection model, in which they propose that two or more cues – e.g., coresidence duration and maternal perinatal association (MPA) - are used by humans to detect kinship and regulate sibling altruism and sexual aversion. As for MPA, we can identify both its operational as well as essential meanings. In the former sense, the presence of MPA implies that the participant and his/her sibling started coresidence from the sibling's birth, while the absence indicates the contrary. In the latter sense, the presence of MPA indicates that a human witnessed his/her mother caring for his/her sibling as a newborn. This approach rejects the existence of a sensitive period. As for MPA-present participants, usually older siblings, the authors observed a linear relationship between participant's age at the beginning of coresidence and incest aversion variables to determine the sensitive period. For MPA-absent participants (usually younger siblings), the authors monitored the partial correlation between the sibling's age at the beginning of coresidence and outcome variables (controlling for coresidence). Since younger siblings usually live with older siblings from birth, in the above two cases, participant's start age and sibling's start age should roughly correspond to the age disparity between the participant and his/her sibling. In conclusion, Lieberman et al. (2007) and Fessler and Navarrete (2004) conducted tests by similar means. However, the former addressed the MPA-present versus MPA-absent distinction regardless of gender, while the latter included gender as an important incest aversion variable.

In general, the WH should be accepted, as it is supported by quite a number of investigations. As for Lieberman et al.'s (2007) general arguments about kin detection and MPA, they indicate a new, promising direction for unraveling the secrets of human incest avoidance, and further empirical study is required. However, is there a sensitive period in human incest avoidance?

Previous research suggests that age three is a critical turning point in the development of personality, cognition, gender identity, and the emergence of self-awareness (Wolf, 1995, pp. 211-213). Piaget argues that children in the third year of life enter a stage of “representational thought” in which their verbal skill develops quickly. Additionally, Bowlby (1982, pp. 356) asserts that the attachment behavior to search for parents' caregiving is usually formed in the first year of life, grows continually, and reaches its peak in the third year. According to Maccoby (1998, pp. 19, 29-30), by the third year of life, girls in various societies spontaneously establish the tendency of playing with samesex partners and avoiding the other sex, and for boys this happens at around age four. Moreover, before the turning points mentioned above, girls and boys are gender-neutral in choosing playmates.

We are unable to find a theory integrating the above findings about the critical period, but we reason that a person may have more intense and frequent interactions with siblings in early cosocialization if the age disparity between him/her and his/her siblings is shorter than about three years. First, under this situation, siblings' active periods of attachment may overlap and they both/all strongly seek caregiving from parents or other caretakers. Second, the siblings may play together and interact with each other more often and intensely in some early period, because at least one of them does not join the samesex group. Since cosocialization constitutes a cue for recognizing kinship, as Lieberman et al. put it, the closer cosocialization and interaction and the intense attachment before around age three may be better cues, and thus have a larger influence on later moral sentiments relating to sibling incest. To conclude, we hypothesize that humans with an opposite-sex sibling less than three years older or younger have stronger aversion to sibling incest than those with an opposite-sex sibling more than three years older or younger.

Testing a sensitive period (e.g., the first three years of life) seems to require a comparison between participants cosocialized with siblings in the first three years of life and participants for which these circumstances did not apply. However, for humans with a younger sibling, usually MPA is present, and MPA is a more potent cue for detecting kinship and avoiding incest (Lieberman et al., 2007), so cosocialization in the first three years is not critical; as for participants with an older sibling, although MPA is absent by Lieberman et. al.'s operational definition, they usually cosocialized with siblings from birth and it would be difficult to find those who did not cosocilaize with siblings in the first three years. By examining whether an age difference matters, we can indirectly test the sensitive period of the first three years of life.

Combining the above reasoning, parental investment theory (Trivers, 1972) and Lieberman et al. (2007)'s argument about MPA, and retaining Lieberman et al.'s operational definition of MPA, we predict the following three outcomes:

1. When MPA is present, as is true when the participant is an older sibling, because MPA is a more potent cue for detecting kinship and avoiding incest, the cue of coresidence in the first three years of life will be used far less or will not be used, and thus we will find that this period does not have any significant effect over later sexual aversion to sibling incest.
2. In females, who are particularly sensitive to the influence of cosocialization in the absence of MPA (true whenever the participant is a younger sibling), cosocialization with a brother in the first three years of life should not have a significant effect in later sexual aversion to sibling incest.
3. For males, when MPA is absent, the effect of the cosocialization in the first three years of life should be significant.

## Materials and Methods

### Participants

There were 1,163 participants, all undergraduates in the Schools of Chemistry, Computer Science, Education, History and Culture, Humanities, Life Sciences, Mathematics and Statistics, and Sociology and Social Work, at the Central China Normal University, Wuhan, P. R. China. To control for possible masking effects of variation in sibling composition, data analysis was restricted to 322 participants with only one opposite-sex sibling and without any other siblings (106 males and 216 females; mean age = 20.47, *SD* = 1.08, age range 17.29 – 23.04).

### Measures

The items used in this study were similar to those of Fessler and Navarrete (2004) and Lieberman et al. (2003). The questionnaire contained questions regarding participant's sex, birthdate, college entrance year, academic major, and sexual orientation, birthdate of sibling(s), and date range of coresidence with sibling(s). If the participant had only one sibling, the questionnaire also asked him/her to indicate certainty of sharing the same biological mother and father, and whether he/she started coresidence with the sibling at the sibling's birth.

The questionnaire told a story about two young adult siblings. They left home to work in a city. To save money, they lived together. Gradually they began to have consensual sex with each other like a couple. Then participants were asked to answer two questions on a 7-point Likert scale: (1) how comfortable they would feel if they happen to interact with the brother and sister; (2) the degree to which they consider the behavior disgusting. The dependent measure SIA (sibling incest aversion) was produced by summing up the ranks on the two items.

Participants reported both their own and their sibling's birthdate (birth year and month), we thereby constructed the primary independent measure SP (sensitive period) - where a score of “1” indicated that the age difference between the participant and his/her sibling was shorter than three years, and a score of “0” indicated that the age difference was more than three years. We produced four other similar dependent variables, SP2, SP4, SP5 and SP6. For SP2, a score of “1” indicated that the age difference between the participant and his/her sibling was less than two years, and a score of “0” indicated that the age difference was more than two years. For MP4, a score of “1” indicated that the age difference was less than four years, and a score of “0” indicated that the age difference was more than four years. For MP5, a score of “1” indicated an age difference of shorter than five years, and a score of “0” indicated an age difference of longer than five years. For MP6, a score of “1” indicated an age difference of less than six years, and a score of “0” indicated an age difference of more than six years. We also established the independent variable Age-Difference to measure the age difference in years between the participant and his/her sibling. We followed Lieberman et al. (2007) to create the independent variable MPA (maternal prenatal association), where a score of “1” indicated that the participant started cosocialization with a sibling at the sibling's birth and is certain they share the same biological mother, and a score of “0” indicated other situations. The independent measure coresidence indexed total length of coresidence between the ages of 0-18 years with the opposite-sex sibling.

### Procedure

Participants completed the questionnaires in classrooms. Before beginning, we told them that there were several sensitive questions and they were free to quit the survey if they felt upset. We also asked beforehand that those who finish the questionnaire first wait quietly until all the participants in the classroom had completed it. All data analyses were conducted using SPSS and STATA. All tests were one-tailed unless otherwise specified.

## Results

*For MPA-present participants, does coresidence with an opposite-sex sibling in the first three years of life significantly affect later sexual aversion to sibling incest?*

**Figure 1.**
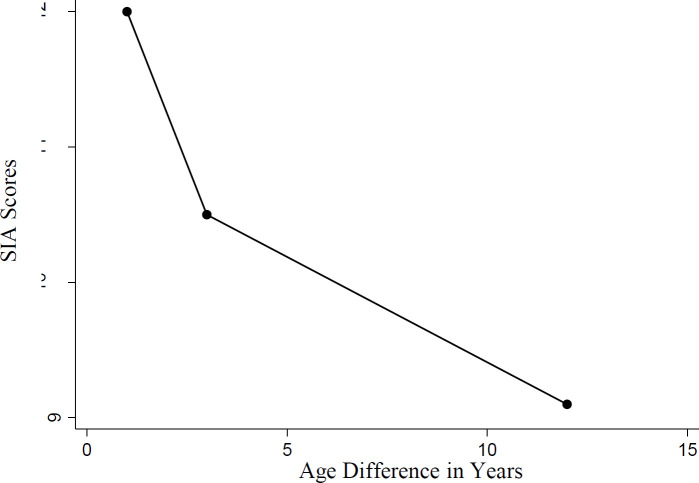
Hypothetical relationship between sibling incest aversion (SIA) and age difference between siblings. If the experience of a period in the life cycle, e.g. the first 3 years of life, has special influence on later sexual aversion, participants whose age difference with their opposite-sex siblings is less than three years may have especially high SIA scores

No. This is the case for both MPA-present males and females. If the first three years of life have significant effects, we should find that those whose age difference with their sibling is shorter than three years are especially averse to sibling incest, and there might be a curvilinear relationship as shown in Figure 1.

There were 31 MPA-present males with only one younger sister. When SIA and Age-Difference were entered into a fractional polynomial regression, the regression model failed to reach significance (*R* ^2^ = .04, *p* = .58). This meant that we were unable to assert that the curvilinear relationships shown in Figure 1 existed.

We divided the 31 MPA-present males into two subgroups—those whose age difference with their younger sister is less than three years and those whose age difference with their sister is greater than three years. The first subgroup included 22 participants and age difference ranged from 1.00 to 2.51 years (*M* = 1.98, *SD* = .39). The second subgroup consisted of nine individuals and age difference ranged from 3.50 to 13.00 years (M = 6.62, *SD* = 2.78). There was no significant difference in the mean SIA of the two subgroups (9.18 vs. 10.22, *p* = .19), and no significant correlation between SIA and SP (*p* = .14). The same pattern emerged when we compared the mean SIA scores for MPA-present females whose age difference with their sibling is: (i) 0-2 years versus not, (ii) 0-4 years versus not, (iii) 0-5 years versus not, and (iv) 0-6 years versus not.

There were 116 MPA-present female participants with only one younger brother. When SIA and Age-Difference were entered into a fractional polynomial regression, the regression model failed to reach significance (*R* ^2^ = .04, *p* = .10). This meant that we were unable to assert that the curvilinear relationship shown in Figure 1 existed.

For the 116 MPA-present females, when the age difference was less than three years, there were 58 participants and age difference ranged from 0.75 to 3.00 years (*M* = 1.80, *SD* = .56); when the age difference was more than three years, there were also 58 participants and the age difference ranged from 3.08 to 14.83 years (*M* = 6.25, *SD* = 2.55). The mean SIA scores of the two subgroups were compared and no significant difference was found (9.24 versus 9.07, *p* = .38). Accordingly, the linear correlation between SIA and SP was not significant (*p* = .38). We also compared the mean SIA scores for MPA-present females whose age difference with their sibling is: (i) 0-2 years versus not, (ii) 0-4 years versus not, (iii) 0-5 years versus not, and (iv) 0-6 years versus not. But we were unable to find any of the above four pairs of subgroups were significantly different in mean SIA score. Accordingly, the correlation between SIA and SP2, SP4, SP5, or SP6, was not significant.

*For MPA-absent females with only one younger brother, does coresidence with a brother in the first three years of life significantly affect later aversion to sibling incest?*

No. For the 100 MPA-absent females, when SIA and Age-Difference were entered into a fractional polynomial regression, the regression model failed to reach significance (*R* ^2^ = .05, *p* = .07). This meant that we were unable to assert that the curvilinear relationships shown in Figure 1 existed.

When the age difference was less than three years, there were 47 participants and age difference ranged from 0.00 to 3.00 years (*M* = 1.98, *SD* = .70); when the age difference was more than three years, there were 53 participants and the age difference ranged from 3.17 to 16.83 years (*M* = 5.80, *SD* = 2.46). The mean SIA scores of the two subgroups were compared and no significant difference was found (9.08 vs. 8.948, *p* = .43). Accordingly, the linear correlation between SIA and SP was not significant (*p* = .43). We also compared the mean SIA scores for MPA-absent females whose age difference with their sibling is: (i) 0-2 years versus not, (ii) 0-4 years versus not, (iii) 0-5 years versus not, and (iv) 0-6 years versus not. But we were unable to find any of the above four pairs of subgroups were significantly different at the mean *SIA* score. Accordingly, the correlation between SIA and SP2, SP4, SP5, or SP6, was not significant.

*For MPA-absent males, does coresidence with a brother in the first three years of life significantly affect later aversion to sibling incest?*

There were 75 MPA-absent males. When SIA and Age-Difference were entered into a fractional polynomial regression, the model approached significance (*R* ^2^ = .07, *p* = .06). For 41 participants, the age difference between the participant and his sister was less than three years, and ranged from 0.00 to 2.92 (*M* = 1.94, *SD* = .62). For 34 participants, the age difference was greater than three years, and ranged from 3.08 to 17.16 (*M* = 6.26, *SD* = 3.33). The mean SIA scores for the two subgroups differed significantly (9.85 vs. 7.15, *p* < .001). Table 1 shows the comparison of mean SIA scores for MPA-absent males whose age difference with their sibling is: (i) 0-2 years versus not, (ii) 0-4 years versus not, (iii) 0-5 years versus not, and (iv) 0-6 years versus not. Among the five pairs of subgroups, for the first three, there was a significant difference in the mean SIA score. This indicated that for MPA-absent males, if their age difference with their sister was less than two, three, or four years, their aversion to sibling incest is greater.

Accordingly, SP, SP2, and SP4 significantly predicted SIA (*r* = .36, *p* < .001; *r* = .21, *p* = .04; *r* = .27, *p* < .01). However, since coresidence duration between the ages of 0-18 years also significantly correlated with SIA (*r* = .32, *p* < .01), and possibly the smaller the age difference, the longer the coresidence duration, might it not be that the effects of SP, SP2, and SP4 are false and function through the coresidence duration? When the effect of coresidence was statistically removed, only the effect size of SP reached significance (partial *r* = .29, *p* = .01, tolerance, 0.85).

For the whole set of male participants, to further test whether only for the MPA-absent males the participants with an age difference of less than 2, 3, or 4 years between them and their sister had higher sibling incest scorers, we created three interaction terms of MPA*SP2, MPA*SP, and MPA*SP4, and entered (SIA, MPA, SP2 and MPA*SP2), (SIA, MPA, SP and MPA*SP), and (SIA, MPA, SP4 and MPA*SP4) into regression equations, respectively.

**Table 1.** Mean sibling incest avoidance (SIA) score for MPA-absent males grouped by age difference with opposite-sex sibling

Age Difference with Sister	Mean SIA Score
Less than or equal to 2 years (*n* = 23)	9.78*
Greater than 2 years (*n* = 52)	8.12
Less than or equal to 3 years (*n* = 41)	9.85***
Greater than 3 years (*n* = 34)	7.15
Less than or equal to 4 years (*n* = 49)	9.35**
Greater than 4 years (*n* = 26)	7.27
Less than or equal to 5 years (*n* = 59)	8.80
Greater than 5 years (*n* = 16)	8.00
Less than or equal to 6 years (*n* = 63)	8.87
Greater than 6 years (*n* = 12)	7.33

* *p* < .05

** *p* < .01

*** *p* < .001

For the first regression (SIA, MPA, SP2, and MPA*SP2), both the model (*F*_3, 102_ = 1.88, *p* = .14) and the betas (MPA β =1.62, *t* = 1.80, *p* = .07; SP2 β = 1.67, *t* = 1.98, *p* = .05; MPA*SP2, β = −2.32, *t* = −1.55, *p* = .12) failed to reach significance. For the second regression (SIA, MPA, SP, MPA*SP), both the model (*F*_3, 102_ = 5.14, *p* < .01, *R* ^2^ = .13) and all the betas (MPA β = 3.08, *t* = 2.55, *p* = 0.01; SP β = 2.71, *t* = 3.63, *p* < .001; MPA*SP, β = −3.75, *t* = −2.54, *p* = .01) were significant. For the third regression (SIA, MPA, SP4, and MPA*SP4), although the model (*F*_3, 102_ = 2.80, *p* = .04, *R* ^2^ = .08) and the betas of MPA (β = 2.73, *t* = 2.04, *p* = .04) and SP4 (β = 2.08, *t* = 2.59, *p* = .01) were significant, the beta of MPA*SP4 (β = −2.77, *t* = −1.76, *p* = .08) was not significant.

Considering the fact that only the beta of the interaction term MPA*SP in the second regression was significant and the *R* ^2^ value of the second model was larger, it seems that the relationship between SIA and SP, instead of SP2 or SP4, depended on MPA. To put it more plainly, it seems that only MPA-absent males whose age difference with their sister is smaller than 3 years had greater sibling incest aversion scores.

## Discussion

Using Lieberman et al.'s (2007) operational definition, we divided participants with only one opposite-sex sibling into four groups: MPA-absent males with one (usually older) sister, MPA-present males with one younger sister, MPA-absent females with one (usually older) brother, and MPA-present females with one younger brother. We found that only for MPA-absent males does a less-than-around-three-years age difference with the sister predict stronger aversion to sibling incest. However, does this finding corroborate the hypothesis of an incest avoidance sensitive period during the first three years of life?

As we reasoned in the Introduction, combining more traditional psychological knowledge from Bowlby, Maccoby, and others, with parental investment theory and Lieberman et al. (2007), if cosocialization with opposite-sex siblings in the first three years of life is especially important for developing later sexual aversion to sibling incest (existence of a sensitive period), we expected the results obtained. But the consistency between the expectation and the main finding does not necessarily support the existence of the sensitive period, and we must consider other possibilities. If the presence of MPA indicates that humans witnessed their mother caring for their siblings as young children, we can extend the initial operational definition of MPA by Lieberman et al. (2007) to include humans whose age difference with their siblings is less than around three years as a MPA-present case. Assuming this, the finding of this study can still be explained by Lieberman et al.'s (2007) hypothesis about the importance of MPA: males whose age difference with their older sister is less than around three years may have witnessed their mother caring for their older sisters as young children, and this constitutes a more potent factor for arousing post-pubescent aversion to sibling incest than cosocialization in the first three years of life. In short, the MPA factor can also explain this study's main finding.

Therefore, we return to our guiding question: Is there a sensitive period? We are willing to leave this question open to be answered by further studies. But what studies? Let's imagine this situation: certain males (e.g., orphans) may live with their older sisters during the ages of 0-18 years, and their sisters were never cared for by a mother-like figure. If we find that those whose age difference with their sisters is less than three years have stronger aversion to sibling incest, we can assert the existence of the sensitive period; otherwise, we can only accept the explanation of MPA. Another approach is to conduct cross-cultural comparative studies. If the duration during which mothers in different societies care for their children varies, and the males whose age difference with their older sister is, e.g., less than three years, have stronger aversion to sibling incest than other males with an older sister, then we can conclude that a sensitive period exists; otherwise, if the age difference between the males, who have stronger aversion to incest, and their older sisters, varies with the duration for which mothers care for their young children, then the role of MPA should be affirmed. For example, if American mothers tend to leave their young children to be cared for by social institutions after the children are two years old, whereas Chinese mothers do so after the children are three years old, if MPA is an important cue, we should expect to find that 1) American males whose age difference with their older sister is less than two years should have stronger aversion than other males with an older sister, and 2) Chinese males whose age difference with their older sister is less than three years should have stronger aversion; if in both cases we find that the age difference of, e.g., three years, indicates a significant difference, then we could more confidently affirm the existence of a sensitive period.
